# The Effects of Low *HER2/neu* Expression on the Clinicopathological Characteristics of Triple-Negative Breast Cancer Patients

**DOI:** 10.31557/APJCP.2020.21.10.3027

**Published:** 2020-10

**Authors:** Mehdi Dehghani, Pedram Keshavarz, Abdolrasoul Talei, Majid Akrami, Sedighe Tahmasebi, Akbar Safaie, Maryam Ghanbari

**Affiliations:** 1 *Hematology Research Center, Department of Hematology and Medical oncology, Shiraz University of Medical Sciences, Shiraz, Iran. *; 2 *Department of Radiology, Medical Imaging Research Center, Shiraz University of Medical Sciences, Shiraz, Iran. *; 3 *Breast Diseases Research Center, Shiraz University of Medical Sciences, Shiraz, Iran. *; 4 *Department of Pathology, School of Medicine, Shiraz University of Medical Sciences, Shiraz, Iran. *; 5 *Department of Internal Medicine, Shiraz University of Medical Sciences, Shiraz, Iran. *

**Keywords:** Triple-negative breast cancer, hormone receptor, HER2/neu, basal-like tumor

## Abstract

**Background:**

Triple-negative breast cancer (TNBC) is an aggressive type of breast cancer (BC), and its diagnosis is associated with negative expression of hormone receptors and HER2/neu. It consists of 10-20% of all BCs diagnosed.

**Methods and materials:**

This study focuses on three groups with different pathology: group one showed complete triple-negative HER2 expression with IHC of BC; groups two and three included patients with ER-, PR-, and HER2^1+^, and ER-, PR-, and HER2^2+^ with a negative FISH test. These three groups were compared from the point of prognosis, which consisted of tumor size, patients’ age, lymphatic, vascular and perineural invasion, organ metastasis, number of lymph nodes involvement, and the survival rate.

**Results:**

A total of 459 TNBC patients were enrolled, of which 268 were placed in the HER2^0^ group, 146 in the HER2^1+^ group, and 45 in the HER2^2+^ group. Distant metastasis and recurrence rate were more common in HER2^0^ patients, but bone metastasis was more common in patients with low HER2 expression. All patients with HER2^0^ had a smaller tumor size at the time of BC diagnosis in comparison to patients in the low HER2 expression group. Patients with HER2^2+^ had less lymphatic and vascular invasion as well as axillary lymph nodes involvement, but larger tumor size at presentation, resulting in a lower rate of recurrence and higher overall survival.

**Conclusion:**

The findings revealed that patients with HER2^2+^ had better outcome in comparison to the patients with HER2^0^ and HER2^1+^. Furthermore, the results showed that many patients with HER2^2+^ expression were not basal-like and had good prognosis amongst TNBC patients.

## Introduction

Breast cancer (BC) is a heterogeneous malignancy amongst females in the USA (Zeichner et al., 2016), and the second cause of cancer death worldwide (first is lung cancer) (Gradishar et al., 2016; DeSantis et al., 2016) Today, there are about 1.5 million BC patients, and 200,000 BC patients are diagnosed every year (Ghislain et al., 2016) While chemotherapy, radiotherapy, and other drug hormonal therapies have led to improvement of BC treatment, almost 25% of patients with primary detectable BC still have recurrence with or without metastasis to other organs, such as bone, liver, lung, etc (Noman et al., 2016). 

Triple-negative breast cancer (TNBC) is a type of BC, which has higher mortality due to its more local recurrence and distant metastasis (Asano et al., 2017), which becomes more resistance to chemotherapy than other types of BC, resulting in shorter survival (Wang et al., 2015; Li et al., 2017) TNBC diagnosis was made possible by the negative expression of hormone receptors and human epidermal growth factor receptor 2 (HER2) neu, which was reported to account for 10-20% of all BC cases (Denkert et al., 2017) TNBC is categorized by the absence of three main biomarkers, i.e. estrogen and progesterone receptors, low expression of HER2 by immunohistochemistry (IHC), and lack of amplification by fluorescence in situ hybridization (FISH) test. The absence of these features means that the hormonal agents and anti-HER2 treatment methods cannot be successful in treating TNBC (Kim et al., 2018; Collignon et al., 2016). The epidermal growth factor receptor (EGFR) activation stimulates cell proliferation and provide protection against apoptosis by activating protein kinase pathways (Li et al., 2017; Kim et al., 2017)

Several methods, such as IHC, chromogenic in situ hybridization (CISH) and FISH are now available to quantify HER2/neu expression.(Goud et al., 2012) Hence, the aim of this study was to compare three groups of TNBCs, namely complete negative HER2 expression by IHC, minimal HER2^(1+)^ expression, and moderate HER2^(2+)^ expression by IHC and the negative FISH test for HER2 amplification. The HER2 negative group included HER2^0^, HER2^1+^, and HER2^2+^, based on differences in the staining intensity and the negative FISH test in the HER2^2+^. This study investigated the possible differentiations in the natural characteristics of TNBC with different low HER2 expressions.

## Materials and Methods

In this retrospective cross-sectional study, a total of 459 cases of triple-negative breast carcinoma were identified from Jun 2002 to Jun 2018 in Breast Disease Clinic, affiliated with Shiraz University of Medical Sciences (SUMS). The clinical and histopathological evaluation of the patients was done by examining a number of pathological features, including age, tumor size, axillary lymph node involvement, and vascular, perineural and lymphatic invasions. Other pathological characteristics, including tumors’ grade, and type of tumor histology, were also evaluated. Distant metastasis was determined and confirmed by history and imaging, including ultrasonography, computerized tomography (CT) scan, and whole-body bone scan. PET-CT scan was used to determine a special case while fine-needle aspiration or needle biopsy was used to confirm the suspicious and difficult cases. Estrogen receptor (ER) and progesterone receptor (PR) status were also noted by IHC. 

We selected only patients with negative estrogen and progesterone hormone, but we divided them into three distinct groups according to their HER2 statuses. Based on IHC, but with negative FISH test for HER2 amplification, the HER2 was determined to be completely negative in the first group, minimal (1+) in the second group, and moderate (2+) positive in the third group. The inclusion criteria were patients with negative estrogen and progesterone hormone, patients with HER2^0^, HER2^1+^, or HER2^2+^ expression, and a negative HER2 amplification by FISH. The exclusion criteria were patients with positive hormone receptors and HER2^3+^ by IHC or HER2^2+^ and a positive amplification by the FISH test. 

*Statistical analysis*

All statistical analysis was performed, using descriptive statistics and independent t-test. Pearson’s Chi-square test was used to calculate the association between the variables. P values < 0.05 were considered to be statistically significant. All statistical analyses were conducted, using SPSS version 21.0.

*Ethics statements*

In this study, all procedures involving human participation were performed in accordance with the ethical standards of the institutional and/or national research committee and the 1964 Helsinki Declaration and its later amendments or comparable ethical standards. The study was approved by the Ethics Committee of Shiraz University of Medical Sciences, Shiraz, Iran (code: IR.SUMS.MED.REC. 1394.s40). 

## Results

In total, 459 TNBC patients joined the study, of which 268 were completely negative for the HER2 expression, 146 patients were HER2^1+^, and 45 patients were HER2^2+^ and a negative HER2 amplification by the FISH test. All patients were followed up from June 2002 through Jun 2018, with a minimum of 16 days to a maximum of 15 years of surveillance and a median of 4.41 ± 2.52 years. The youngest patient, aged 24 was in the HER2^0^ group while the oldest one aged 81 was in the HER2^1+^ group. The mean age characteristics of the three distinct groups are presented in Table 1. 

The evaluation of the histopathological characteristics of the patients and a comparison of the lymphatic invasion showed a minimal higher rate of invasion in the HER2^0 ^and HER2^1+^ groups, but the difference was not statistically significant.(P = 0.6) A comparison of the extent to which the right- and left-side breast were affected by the tumor showed that the right-side breast was affected more 66.6% (n = 30) by the tumor in the HER2^2+^, but the difference was not statistically significant (P = 0.1). Therefore, it can be stated that in all three groups, both sides of the breast were equally affected by tumor. The type of surgery was affected by the patient’s preference, size of the tumor, and the tumor stage. A comparison between groups revealed that patients in the HER2^1+^ group had undergone more breast conservation surgery; however, the difference was not statistically significant (P = 0.7) (Table 1).

The stage of disease at presentation was affected by the tumor size, but the lymph nodes involvement was the most significant factor in BC prognosis. The patients were also divided into three groups based on their tumor size, i.e. patients with tumor size less than 2 cm were allocated in the first group, patients with tumor sizes of equal or more than 2 cm, and equal or less than 5 cm in the second group, and patients with tumor size more than 5 cm in the third group. The results indicated that in the HER2^0^ group, 26.5% (n = 71) of patients belonged to the first group (tumor size < 2cm), 58.6% (n = 157) to the second group (tumor size between 2 and 5cm), and 14.9% (n = 40) to the third group (tumor size > 5cm). It appeared that the overall size of tumor in the HER2^0^ group was smaller at BC presentation. On the other hand, tumor sizes of more than 5 cm were slightly more common in the HER2^1+^ and HER2^2+^ groups, with 17.8% (n = 22) and 22.2% (n =10) of patients identified to have tumor sizes more than 5 cm in these two groups, respectively. (Table 1) Invasive ductal carcinoma (IDC) was the most predominant subtype of BC in all three types of cancer. About 69% (n = 185) of IDC was found in HER2^0^ and a total of 72.3% (n = 332) in all the groups together while the medullary carcinoma was prominent in the HER2^0^ subtype with about 16.7% (n = 45) of cases, and 13.9% (n = 64) of the total patients in all the groups. The lobular and other pathologic subtypes are not common in TNBC. 

After tumor size, the most important factor in the staging of BC was lymph nodes involvement. The patients were also categorized according to the numbers of lymph nodes involvement either by sentinel lymph node biopsy or axillary lymph nodes dissection. The first group consisted of patients with less than three lymph nodes involvement, the second group patients with equal and more than three, and equal and less than ten lymph nodes involvement, and the third group patients with more than ten lymph nodes involvement. The most lymph nodes involvement was observed in the HER2^0^ group with 92 patients and 3(3%) of the HER2^0^ patients had more than 10 lymph nodes involvement, which was the worst prognosis and an ominous factor for local recurrence and distant metastasis. In the HER2^2+^group, we observed that most of the patients had none or less than three lymph nodes involvement. In comparing the involvement of the axillary lymph nodes, especially in patients with more than ten lymph nodes, which is associated with a shorter lifespan and we observed that the lowest rate is in the HER2^2+ ^group and also the lowest mortality rate is seen in this group, although it is not much different in terms of statistics (Table 2).

With regard to the distant metastasis, 56 out of 459 patients had distant metastasis and the most common sites were lung, liver, and bone, respectively. The distant metastasis rate was slightly more common in the HER2^0 ^group 14.9% (n = 40) than in the HER2^2+^ 11.1% (n = 5) and HER2^1+^ 7.5% (n = 11) groups. Also, brain metastasis was slightly more common in the HER2^0^ group, but the differences were not significant. The following results were obtained based on the local recurrence: 21.1% (n = 53) of patients in the HER20, 17.3% (n = 24) in the HER2^1+^ group, and 11.4% (n = 5) in the HER2^2+^ group had local recurrence. This meant that the local recurrence rate was slightly higher in the HER2^0^ patients compared to HER2^1+^ and HER2^2+^ groups (Table 3).

In the study of tumor invasion, we observed the lowest rate of vascular and lymphatic invasion, as well as the lowest rate of metastasis to the liver and brain, and multiple metastases to various organs are seen in the HER2^2+^ group (Table 4).

**Figure 1:**
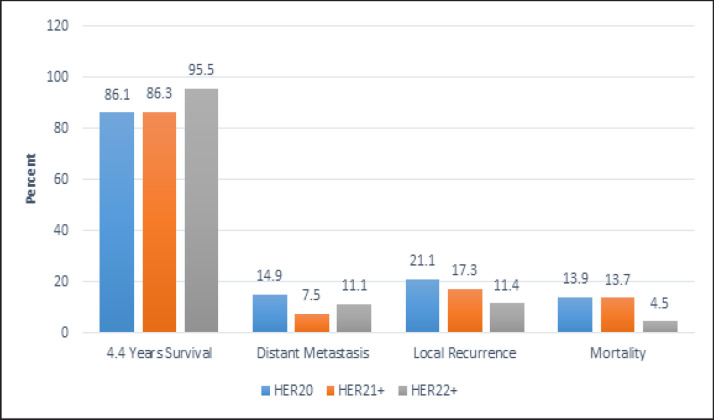
Comparing Survival Rate, Distant Metastasis, Local Recurrence and Mortality Rate

Selecting radiotherapy as a therapeutic modality is multifactorial, including tumor size, axillary lymph nodes involvement and the type of surgery. In this study, no significant difference was observed between the three study groups with respect to radiation. Majority of the patients, 84.2%, 85%, and 83.8%, in the HER2^0^, HER2^1+^, and HER2^2+^ groups, respectively, received radiation. From June 2002 to June 2018, 86.1% (n = 231) of the HER2^0^ patients were alive and the mortality rate in this group was 13.8% (n = 37); in the HER2^1+^ group, 86.3% (n = 126) were alive during the same period and the mortality rate was 13.6% (n = 20) for this group. In the HER2^2+^ group, only 4.4% (n = 2) of patients were died and 95.5% (n = 43) were still alive. As the above findings indicate, the mortality rate was very low in the HER2^2+^ group, but the difference between the groups was not statistically significant (P = 0.2) (Figure1) 

**Table 1:** The Clinical Characteristics of the Patients and Tumor Samples Used in the Study

Characteristics	N	HER2^0^ N (%)	HER2^1+^ N (%)	HER2^2+^ N (%)	P
Age					
Mean age ± St. deviation		46.5±12.14	46.3±8.09	48.6±6.83	N.S
Tumor size (1)*					
T < 2cm	134	71 (26.5)	47 (32.2)	16 (35.6)	N.S
2cm ≤ T ≤ 5cm	249	157 (58.6)	73 (50)	19 (42.2)	
T > 5cm	76	40 (14.9)	26 (17.8)	10 (22.2)	
Lymph nodes invasion (1)**					
N < 3	83	67 (25)	2 (1.3)	14 (31.1)	N.S
3 ≤ N ≤ 10	32	22 (8.2)	9 (6.1)	1 (2.2)	
N > 10	4	3 (3)	1 (0.7)	0	
without lymph node involvement	340	176 (65.6)	134 (91.7)	30 (66.6)	
Side of invasion ***					
Right	234	135 (50.3)	69 (47.2)	30 (66.6)	N.S
Left	225	133 (49.6)	77 (52.7)	15 (33.3)	
Surgery ****					
BCS	267	156 (58.9)	87 (59.6)	24 (53.3)	N.S
MRM	192	112 (41.8)	59 (40.4)	21 (46.6)	
Total	459	268	146	45	N.S

BCS, Breast conserving surgery; MRM, Modified radical mastectomy; N.S, not significance; * P value, 0.2; ** P value, 0.5; *** P value, 0.1; **** P value, 0.7

**Table 2:** The Rate of Involvement of the Lymph Nodes in the Three Separate Groups

Cancer type	Mean age	Lymph nodes involvement less than 3	Lymph nodes involvement (3-10)	Lymph nodes involvement more than 10	Survival (Alive)in 4.4 years
HER2^0^	46.5	77.80%	23.90%	3.30%	86.10%
HER2^1+^	46.3	71.40%	25.70%	2.90%	86.30%
HER2^2+^	48.6	93.30%	6.70%	0	95.50%

**Table 3:** The Histopathological Characteristics of the Patients who Took Part in the Study

Characteristics	N	HER2^0^ N (%)	HER2^1+^ N (%)	HER2^2+^ N (%)	P
Histopathology					
Insitu	9	4 (1.5)	3 (2.1)	2 (4.4)	N.S
IDC	332	185 (69)	114 (78)	33 (73.3)	N.S
ILC	4	3 (1.1)	1 (0.7)	0	N.S
Mucinous	3	2 (0.7)	1 (0.7)	0	N.S
Medullary	64	45 (16.7)	12 (8.2)	7 (15.5)	N.S
Papillary	3	3 (1.1)	0	0	N.S
Others	44	26 (9.7)	15 (10.2)	3 (6.6)	N.S
Total	459	268	146	45	N.S
Local recurrence *					
Yes	82	53 (21.1)	24 (17.3)	5 (11.4)	N.S
No	352	198 (79.8)	115 (82.7)	39 (88.6)	
Metastasis **					
Yes	56	40 (14.9)	11 (7.5)	5 (11.1)	N.S
No	403	228 (85.1)	135 (92.4)	40 (88.8)	

IDC, Invasive ductal carcinoma; ILC, Invasive lobular carcinoma; N.S, Not significant; * P value, 0.2; ** P value, 0.6.

**Table 4:** Tumor Invasion and Metastasis to Various Organs

	Lymphatic invasion	Vascular invasion	Perineural invasion	Tumor size more than 5cm	Liver metastasis	Lung metastasis	Bone metastasis	Brain metastasis	Multiple sites of metastasis	Total case
HER2^0^	14.20%	2.50%	5%	14.90%	3.40%	4.70%	2.60%	1.15	3.10%	268
HER2^1+^	16.50%	2.40%	3.40%	17.80%	1.40%	0	1.40%	0.70%	3.50%	146
HER2^2+^	9.80%	0	9.85	22.20%	0	6.70%	2.20%	0	0	45

## Discussion

Breast cancer (BC) is referred to as type of mixed tumors with different actions, prognosis, and difficult to treat (Noman et al., 2016) Many studies reported that about 15% of BC patients are TNBC positive. This type of cancer is more aggressive than other BC types and has poorer prognosis compared to other BC types (Speers et al., 2017; Kucukzeybek et al., 2018) Recent studies revealed that BC heterogeneity extends to the classic IHC-based subtypes of ER, PR, and HER2 receptors.(Gradishar et al., 2016; Kurozumi et al., 2016) Nowadays, new treatment methods are suggested for TNBC according to new biomarkers and gene expression profile (Burstein et al., 2015; Sarin et al., 2016).

Our results suggest that the three groups in the present study were close to each other with regard to the investigated variables. For some parameters, such as metastasis, recurrence, and tumor size, the HER2 status did not significantly change in the BC prognosis. Regarding the local recurrence, the results showed a 21.1% for the HER2^0^ group and 17.3% for the HER2^1+^ group vs. 11.4% for the HER22+ group. With regard to the metastasis to solid organs, the findings showed 24.3% metastasis in the HER2^0^ group vs. 8.9% in the HER2^1+^ group and 7.5% in the HER2^2+^ group. More solid organ metastasis was observed in the HER2^0^ group, but was not significantly different between groups. A comparison of the three groups regarding the tumor size and axillary lymph nodes involvement indicated that there was a larger tumor size at presentation in the HER^2+^ patients, but more axillary lymph nodes involvement was observed in the HER2^0^ group. These findings show more aggressive behavior in HER2^0^ than in HER2^2+^ patients.

A second comparison was made to gain more specific data about the HER2 status role in the prognosis of BC patients. The results indicated that HER2^2+^ patients had a higher survival rate, a lower rate of lymph nodes involvement, less lymphatic invasion, and a lower rate of local recurrence. Both the current study and the study by Plasilova et al. revealed that HER2^2+^ was a protective factor in BC patients. In other words, many HER2^2+^ patients were not affected by the basal-like tumor.(Plasilova et al. 2016) Regarding the lymph node involvement, the findings revealed that the HER2^2+^ status in BC meant that more lymph nodes were involved. It was also found that HER2^2+^ patients had more perineural invasion, but less lymphatic invasion. All three groups had the same distribution regarding the three categories of tumor size. To be more specific, there were more patients in all the three groups who were classified in the category of 2-5 cm, but the number was higher for the patients in the HER2^2+^ group. Contrary to the expectation, TNBC group had a smaller tumor size (14.9% in the HER20 group, 17.8% in the HER2^1+^ group, and 22.2% in the HER2^2+^ group had a tumor size more than 5 cm). Other studies have shown that even small tumors seem to have a higher recurrence rate in the HER2^0^ group, suggesting a correlation between larger tumor sizes and the increase of HER2 receptor status, and also between larger tumors and the HER2^2+^ group.(Amar et al. 2010; Kaplan, Malmgren, and Atwood 2009) However, metastatic BC was more common in TNBC, and there was no significant difference between the HER2^1+^ and HER2^2+^ groups regarding metastatic BC. In another study, Schmidt et al. had divided TNBCs into three subtypes, i.e. HER2/neu score 0, score 1, and score 2. The results of their study revealed that TNBC patients with a HER2/neu score of 0 had a significantly poorer outcome regarding disease-free survival.(Schmidt et al., 2016) Therefore, the identification of HER2^2+^ in TNBC patients can play a significant role in the prognosis of this disease, which is a contribution of this study.

According to the tumor staging system and the evaluation of the tumor size, lymph nodes involvement, and distant metastasis, it became clear that patients with HER20 had more basal-like behavior and a more aggressive type of tumor; whereas patients with HER^2+ ^had a less aggressive tumor and mostly were not basal-like tumor. Equally important, the results of this study showed that both the rate of recurrence and metastasis rate were higher in the TNBC patients (just bone metastasis was higher in the HER2^1+^ or HER2^2+ ^with negative FISH); however, the TNBC patients generally had a smaller tumor size. 

Several biomarkers, including cytokeratin 5/6, EGFR, and negative staining for both ER and HER2, which are highly sensitive to basal-like BC might account for these differences between the basal-like and triple-negative tumors. Additional biomarkers, such as cytokeratin 14 or 17, p53 stabilization, and higher mitotic indices, are associated with worse survival in triple-negative tumors. One potential strategy for confirming the basal-like tumors is the evaluation of the 50-gene expression profile to determine the molecular subtype; however, this methodology is not practical in many centers. It should be noted that many new molecular targets, including phenotypic and genomic analysis, are prognostic and might have therapeutic effects on TNBC (Johnstone et al., 2020; Shan et al., 2019) Irrespective of the new molecular and genomic markers that have significant effect on the prognosis of BC, a more practical strategy for determining the basal-like TNBC and its prognosis is the use of HER2 by IHC to identify the protein expression surrogates for the basal-like gene signature (Toft and Cryns, 2011)

In conclusion, to conclude, it can be stated that HER2^2+^ status makes the TNBC cells are less aggressive and result in a better prognosis. More research should be conducted to further our understanding the TNBC characteristics and its prognosis so that we can develop better treatment methods and easier ways to increase the survival rate of patients with this type of BC. 
